# Late Infected Left Ventricular Pseudoaneurysm Following LVAD Explantation After Trauma

**DOI:** 10.1016/j.jaccas.2025.104008

**Published:** 2025-06-04

**Authors:** Mouna Mohib, Frederic Vanden Eynden, Isabelle Huybrechts, Charles Dehout

**Affiliations:** a Department of Intensive Care, Hôpital Universitaire de Bruxelles-Hôpital Erasme, Brussels, Belgium; b Department of Cardiac Surgery, Hôpital Universitaire de Bruxelles-Hôpital Erasme, Brussels, Belgium; c Department of Anesthesiology, Brussels University Hospital (H.U.B.) Erasme, Brussels, Belgium

**Keywords:** anesthesia, cardiac assist devices, ventricular septal defect

## Abstract

We report a rare case of an infected left ventricular pseudoaneurysm arising late after left ventricular assist device (LVAD) explantation. The condition was triggered by minor thoracic trauma and presented as a pulsatile mass. This case highlights diagnostic challenges and therapeutic strategies for late-onset complications post-LVAD explantation.

## Graphical Abstract

**Figure:**
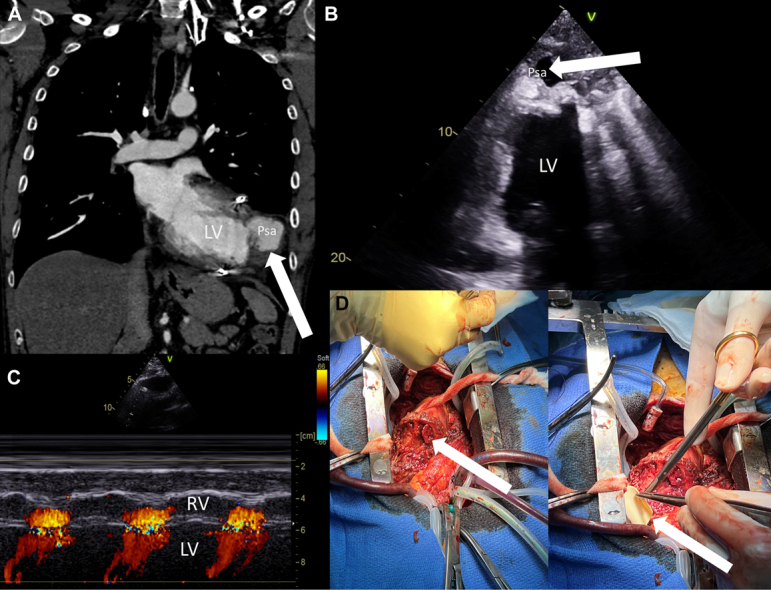

## Case Presentation

A 40-year-old man with a history of alcoholic dilated cardiomyopathy managed with an assist device (HeartWare Inc ventricular assist device) for 7 years underwent left ventricular assist device (LVAD) explantation after partial cardiac recovery. Two years postexplantation, he presented with a pulsatile left thoracic mass that had progressively developed over 6 weeks after minor trauma (Video 1).

**Take-Home Messages**

• Late-onset infected left ventricular pseudoaneurysm can develop after LVAD explantation, even years later and after minor trauma.
• Early imaging, surgical intervention, and targeted antibiotic therapy are essential for successful management of infected postexplant pseudoaneurysms.

**Figure 1:**
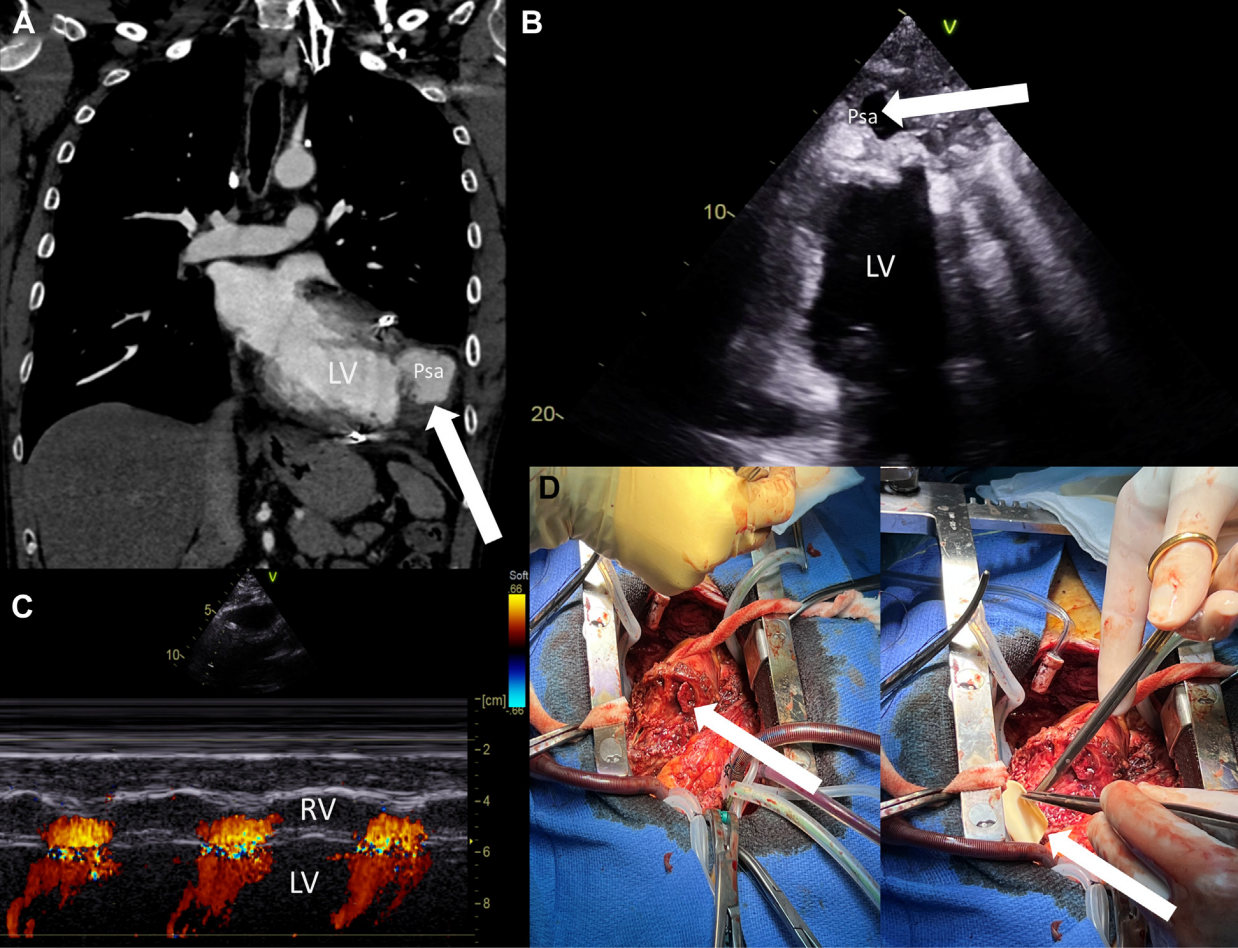
Multimodal Imaging and Intraoperative Findings of a Left Ventricular Pseudoaneurysm Following LVAD Explantation (A) Chest computed tomography showing the pseudoaneurysm (white arrow) of the left ventricular wall. The pseudoaneurysm pocket measured 52 mm in the largest meter and 34 mm in its orthogonal diameter with a height of 38 mm. (B) Transthoracic echocardiography revealing a pseudoaneurysm (white arrow) at the apex of the heart. (C) M-mode and color Doppler highlighting the defect in the interventricular septum, causing a left-to-right shunt at the level of the interventricular communication. (D) Surgical view via sternotomy. The white arrow on the left shows the defect in the interventricular septum. The white arrow on the right shows the patch used for closing the interventricular communication.

Computed tomography revealed a 52 × 34 × 38-mm pseudoaneurysm at the left ventricular lateral wall, proximal to the prior LVAD implantation site (Figure 1A). Echocardiography confirmed the pseudoaneurysm encircling the seventh rib's anterior arch (Figure 1B, Video 2). It also revealed an iatrogenic ventricular defect, present since LVAD implantation, that was still documented and even more pronounced (Figure 1C).

Surgical repair was performed using a median sternotomy with cardiopulmonary bypass. The pseudoaneurysm and ventricular septal defect were repaired successfully with sutures and a synthetic pericardial patch, respectively (Figure 1D). The patient recovered uneventfully, but *Staphylococcus epidermidis* and *Staphylococcus warneri* were cultured intraoperatively without any preoperative signs or symptoms of infection. The patient was treated with a 6-week course of cefazolin and rifampicin. At 3 months, the patient demonstrated full functional recovery without recurrence.

## Discussion

LVADs have transformed the management of advanced heart failure, particularly as bridges to transplantation but are sometimes used as bridges to recovery.^1^ In such instances, safe removal of the pump, although technically demanding, avoids long-term complications related to the device.^2^ Reports document pseudoaneurysms during LVAD support, but this is the first documented case of an infected left ventricular pseudoaneurysm triggered by infection and trauma occurring years after device explantation.^3^

Surgical intervention remains the mainstay of treatment for left ventricular pseudoaneurysm, and infection management demands pathogen-specific antimicrobial regimens. This case underscores the need for vigilance in monitoring patients post-LVAD explantation, even years later, for delayed complications such as infection-induced pseudoaneurysms.

## Funding Support and Author Disclosures

The authors have reported that they have no relationships relevant to the contents of this paper to disclose.

## Appendix

Video 1 Pulsatile left laterothoracic mass gradually evolving over the past month and a half after a mechanical fall onto the left thorax. A progressive mass is seen with a prominent murmur on auscultation and a pulsatile compressible palpation regarding the sixth and seventh ribs.

Video 2 Transthoracic echocardiography revealing a pseudoaneurysm at the apex of the heart demonstration of circulating flow within the pseudoaneurysm.
